# Case of Sudden Death from a Morbid Affection of the Brain; with the Appearances on Dissection

**Published:** 1804-11-01

**Authors:** John Clarke, Thomas Bradley

**Affiliations:** Physician to the Assylum for Female Orphans; Physician to the Assylum for Female Orphans


### Case of sudden Death from a morbid Affection of the Brain; with the Appearances on Dissection. By John Clarke, M. D. and Thomas Bradley, M. D. Physicians to the Assylum for Female Orphans.

THE following Case is recorded as one among many, which prove that the brain, though an organ of great importance to the functions of life, admits of very considerable alterations in its structure, and for a great length of time, without the existence of any symptoms which excite the attention of the patient.

A. B. aged 11, an orphan in the Assylum, had enjoyed such good health during the whole time of her residence there, as never to have been admitted as a patient into the Infirmary on any account; and upon inquiry, it does not appear that she had ever made any complaint to any of the other children, of any pain or sickness. On the contrary, on the morning when she was attacked with the disease, of which she died, she had been playing with the other girls, and in very good spirits.

On Thursday, October 4, whilst she was in the school, employed in needle-work, she was suddenly seized with a most violently acute pain in the pit of the stomach, so that she was not able to walk without assistance. She was immediately taken to the Infirmary, (a distance of about sixty yards) and in the space of a very few minutes she expired.

On

On the following morning, October 5, her body was examined, under the direction of Mr. Howard, surgeon to the Institution, and in the presence of the writers of this Report.

The attention was, of course, first directed to the cavity of the abdomen, where she had complained of pain; but on a minute inspection, all the contents of this cavity were found perfectly sound.

The thorax was next subjected to examination, but no disease of any part was found there, both the lungs and heart being quite in a healthy state.

The contents of the skull were then exposed; and the first circumstance which attracted attention was the unusual vascularity of the upper surface of the pia mater, the vessels upon which were much larger than they are usually found. At the anterior part of the brain, the division between the two hemispheres, upon taking off the dura mater, was less perfect than it is commonly found; and at this part, between the tunica arachnoides and pia mater, there appeared a quantity of water, which escaped upon making a small puncture with a scalpel. The substance of the brain did not exhibit any unusual appearance; but upon cutting into the lateral ventricles, there was found a much larger quantity of water than is natural to those cavities, yet not enough to produce any probable effects from pressure, or distention, and there was no extravasation of blood either on the surface of the brain, or in the ventricles.

The brain was then carefully removed, for the purpose of inspecting the under surface. The general appearance was natural; but under the middle lobe of the left side there was a cyst, (which contained more than half an ounce of a transparent fluid) one side of which, to wit, the inferior, was apparently formed by the membranes. The upper was formed by the substance of the brain itself, which at this part was hollowed or indented, and was very white, of the colour of the medullary substance of the brain.

The rest of the brain, and the cerebellum, appeared to be in a healthy state.

There can be very little doubt that the sudden death of the patient may be fairly attributed to this cause. Yet the remarkable circumstance respecting it is, that although the disease must have been of very long standing, as is proved by the absorption or compression of the brain at the inferior part of the middle lobe, where the cyst was formed, yet no symptom indicating any disease of the head had existed existed till within a very few minutes before her death; even then, the sensation of pain was referred to the stomach, and not to the head, so that it might perhaps even be doubted by some, whether, although the derangement of the structure of the brain above described was found upon dissection, this was in fact the cause of her death.

We are of opinion, however, that such doubts will be removed, when it is remembered that there is a very close connexion by sympathy between the brain and the stomach, and that diseased affections of the former very commonly produce effects on the latter. The sickness which takes place in apoplexy, in injuries of the brain from fracture, extravasation, &c. are decisive as to this. But perhaps there is no circumstance in which this more strongly appears than in puerperal convulsions, which are most frequently excited by organic affection of the brain as an occasional cause; of the approach of which, violent pain of the stomach is sometimes the only symptom. Why, in this instance, the effect which terminated in the death of the patient was produced at that time, or why some symptoms of the existence of so considerable a diseased alteration of so important a part, had not sooner appeared, we do not undertake to explain; but we take occasion to observe, that a case fell within the knowledge of one of the reporters of this case, in which a woman died of a sudden and unexpected attack of puerperal convulsions; and upon the examination of her body after death, a very considerable suppuration of the plexus choroides appeared, which must necessarily have existed before the attack of the convulsions, yet there had been no symptom indicating the existence of any disease of the brain beforehand.

*London, October* 10, 1804.

